# Thymic stromal lymphopoietin (TSLP) inhibits human colon tumor growth by promoting apoptosis of tumor cells

**DOI:** 10.18632/oncotarget.7614

**Published:** 2016-02-23

**Authors:** Wenjie Yue, Yuli Lin, Xuguang Yang, Bingji Li, Jie Liu, Rui He

**Affiliations:** ^1^ Department of Immunology, School of Basic Medical Sciences, Key Laboratory of Medical Molecular Virology of Ministries of Education and Health, Shanghai, 200032, People's Republic of China; ^2^ Department of Digestive Diseases, Huashan Hospital, Shanghai, 200032, People's Republic of China; ^3^ Biotherapy Research Center, Fudan University, Shanghai, 200032, People's Republic of China

**Keywords:** TSLP, colon cancer, apoptosis, TSLPR, caspase3

## Abstract

Thymic stromal lymphopoietin (TSLP) has recently been suggested in several epithelial cancers, either pro-tumor or anti-tumor. However, the role of TSLP in colon cancer remains unknown. We here found significantly decreased TSLP levels in tumor tissues compared with tumor-surrounding tissues of patients with colon cancer and TSLP levels negatively correlated with the clinical staging score of colon cancer. TSLPR, the receptor of TSLP, was expressed in all three colon cancer cell lines investigated and colon tumor tissues. The addition of TSLP significantly enhanced apoptosis of colon cancer cells in a TSLPR-dependent manner. Interestingly, TSLP selectively induced the apoptosis of colon cancer cells, but not normal colonic epithelial cells. Furthermore, we demonstrated that TSLP induced JNK and p38 activation and initiated apoptosis mainly through the extrinsic pathway, as caspase-8 inhibitor significantly reversed the apoptosis-promoting effect of TSLP. Finally, using a xenograft mouse model, we demonstrated that peritumoral administration of TSLP greatly reduced tumor growth accompanied with extensive tumor apoptotic response, which was abolished by tumor cell-specific knockdown of TSLPR. Collectively, our study reveals a novel anti-tumor effect of TSLP via direct promotion of the apoptosis of colon cancer cells, and suggests that TSLP could be of value in treating colon cancer.

## INTRODUCTION

Thymic stromal lymphopoietin (TSLP), as an epithelial cell derived cytokine, plays an important role in barrier immunity by mainly acting on immune cells such as dendritic cells, mast cells and CD4^+^T cells [1, 2]. TSLP exerts its biological activities by binding to a heterodimeric receptor consisting of the IL-7Rα and the TSLP receptor (TSLPR) [1, 3, 4]. It is widely accepted that TSLP plays an important role in initiation and maintenance of Th2 cell-mediated allergic inflammation [5, 6]. Increased TSLP expression is detected and contributes to the immunopathologies with dysregulated Th2 cell-type cytokine production including allergic asthma and atopic dermatitis [7–9]. In contrast to the inducible expression of TSLP in airway and skin, TSLP is expressed constitutively in intestinal epithelial cells (IECs) from healthy subjects, with its highest levels in colonic epithelial cells [10]. Moreover, decreased TSLP production was found in mucosal biopsies from patients with inflammatory bowel disease (IBD) [11]. TSLP-TSLPR interaction has been shown to protect DSS-induced colitis by either inhibiting Th1 cell-type cytokine production or promoting mucosal healing [10, 12]. These data suggest that the role of TSLP-TSLPR interaction in tissue inflammation could be context-dependent.

TSLP recently has emerged as a contributing factor to some cancers. Increased TSLP was detected in some tumors such as breast cancer and pancreatic cancer, both of which are associated with Th2-related chronic inflammation [13–15]. TSLP produced by either tumor cells or cancer-associated fibroblasts was important for Th2 cell differentiation and intratumoral infiltration, allowing a permissive environment for the growth and metastasis of these tumors [13–15]. The increasing interest in the role of TSLP in cancer has focused on the immune-regulatory effect of TSLP on different immune cells. However, functional TSLPR has been recently reported to be expressed on some non-hematopoietic cells such as human intestinal epithelial cells and airway smooth muscle cells [12, 16], suggesting a role of TSLP in non-immune cells. Colon cancer is the third most common cancer worldwide and the fourth most common cause of death [17]. A recent human study demonstrated that Th2 cells and related cytokines are not associated with the prognosis of patients with colon cancer [18]. To our knowledge, there is so far no study investigating TSLPR expression by colon cancer cells that originate from intestinal epithelial cells, the direct effect of TSLP on tumor cell biology, and the underlying molecular mechanism.

In this study, we examined the expression of TSLP at both mRNA and protein levels in colon tumor and macroscopically uninvolved surrounding tissues from surgical specimens, and TSLPR expression in human colon cancer cells and tumor tissues. We then evaluated the effect of TSLP on the growth of colon cancer cells *in vitro* and *in vivo*. Our study revealed a novel anti-tumor role of TSLP by directly acting on human colon cancer cells through TSLPR signaling to induce their apoptosis.

## RESULTS

### Locally decreased TSLP expression in human colon tumors

**Figure 1:**
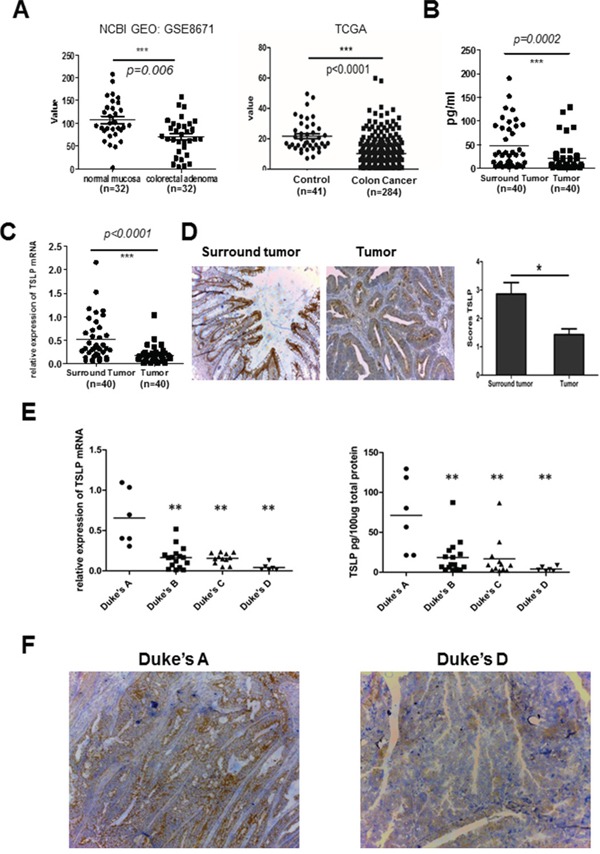
Decreased tumoral TSLP expression in human colon cancer **A.** TSLP expression in colon adenomas and normal mucosa by using two sets of public data from the GEO database (NCBI GEO: GSE8671) and TCGA cancer genome. **B and C.** TSLP mRNA expression was examined by quantitative real-time RT-PCR (qRT-PCR) analysis **(B)** and TSLP protein levels were measured by ELISA **(C)** in tumor and tumor-surrounding tissues from surgical specimens of 40 colon cancer patients. **D.** Representative photomicrographs of immunohistochemical (IHC)-staining of TSLP in tumor and tumor-surrounding tissues (100X magnification) and quantitiative analysis of the intensity of TSLP staining. *P<0.05. **E.** The analysis of TSLP mRNA and protein levels in tumor tissues from colon cancer patients according to the criteria of Duke's classification. **F.** Representative photomicrographs of IHC-staining of TSLP in tumor tissues from colon cancer patients in stage A and stage D (100X magnification). Individual tissue samples are represented as dots in a,b,c.*P<0.05 **P<0.01.

Elevated TSLP expression has been reported in some types of tumor, such as breast and pancreatic tumor, which promotes tumor growth and positively correlates with the presence of metastasis and negatively with prognosis [7, 13]. To assess TSLP expression levels in colon cancer, we first compared the gene expression of TSLP in colon adenomas with that in normal mucosa from the same individuals by using well-annotated NCBI GEO datasets [19] (Figure 1A). Interestingly, we found that TSLP mRNA expression significantly decreased in colon adenomas. To further confirm our finding, we collected tissue samples of the tumor and tumor-surrounding areas from 40 patients with colon cancer who underwent surgical resection in Huashan Hospital, Fudan University. The clinic characteristics of these patients were shown in Supplementary Table S1. We compared TSLP expression in tumor and macroscopically uninvolved surrounding tissues from surgical specimens at both mRNA and protein levels by qRT-PCR and ELISA assay of tissue homogenates, respectively. Significantly decreased TSLP mRNA expression and an approximate two-fold decrease in the average protein levels of TSLP (47.54±7.66 vs 22.46±5.09, n=40, p<0.01, mean±SEM) were detected in tumor tissues compared with tumor-surrounding tissues (Figure 1B and 1C). Furthermore, TSLP immunostaining showed that the epithelium of normal mucosa in tumor-surrounding tissues was positively stained with TSLP (Figure 1D), which is consistent with the previous findings that colon epithelial cells constitutively express TSLP [18]. In contrast, a much lesser extent of TSLP-positive staining was detected in the tumor tissues (Figure 1D), which was consistent with the results of ELISA assay.

We next investigated whether decreased TSLP expression was associated with the clinical severity. To this end, we compared TSLP expression levels in the tumor tissues from the above totally 40 patients according to their Duke's stage from stage A to stage D, which is widely used in clinic to evaluate the severity and the prognosis of colon cancer. The tumor tissues from patients with stage A colon cancer had significantly higher TSLP expression at both mRNA and protein levels than those from patients with stage B, C and D colon cancer (Figure 1E). The lowest expression levels of TSLP were found in the tumor samples from patients with stage D colon cancer that usually predicts remote metastasis and poor prognosis (Figure 1E) [20], although TSLP expression levels were comparable between tumor tissues from stage B and stage C patients (Figure 1E). This was further confirmed by TSLP immunostaining of tumor sections from patients with stage A and stage D colon cancer showing that tumor tissues from stage A patients had much more TSLP-positive staining than those from stage D patients (Figure 1F). Taken together, our data demonstrated that TSLP expression was down-regulated in the tumor tissue of patients with colon cancer, moreover, TSLP expression levels may negatively correlate with the severity of colon cancer.

### Expression of TSLPR in human colon cancer

**Figure 2:**
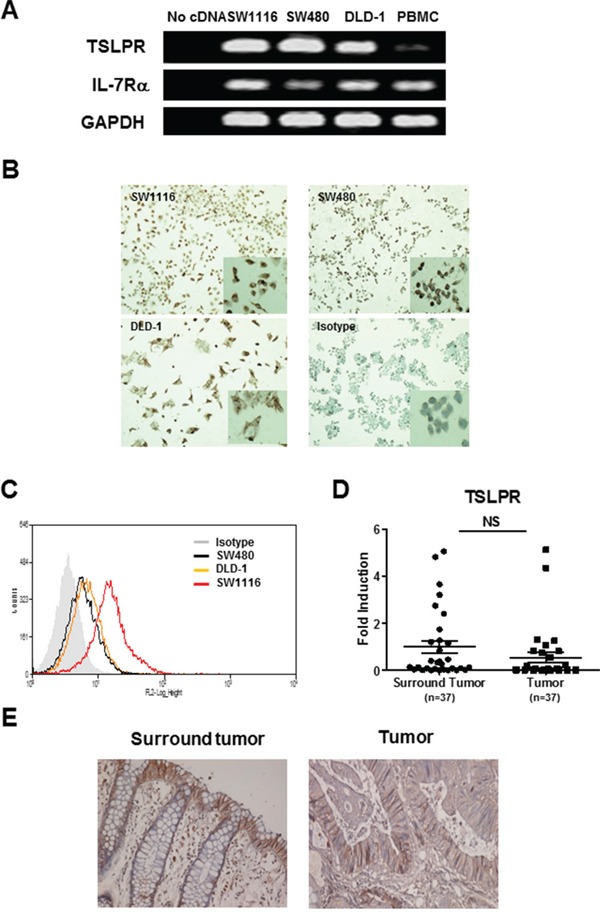
TSLPR expression in colon cancer cells **A.** TSLPR mRNA expression was examined by conventional RT-PCR analysis in colon cancer cell lines, SW1116, SW480 and DLD-1. PBMCs were used as a positive control. **B and C.** TSLPR expression in colon cancer cell lines was detected by immunostaining (100X magnification, inserted: 200X magnification) **(B)** and was analyzed by flow cytometry **(C)**. **D and E.** qRT-PCR analysis of TSLPR mRNA expression **(D)** and representative photomicrographs of IHC-staining of TSLPR (100X magnification) **(E)** in tumor and tumor-surrounding tissues, respectively. Individual tissue samples are represented as dots in d. NS=no significant.

In spite of extensive studies of TSLP function in immune cells, recent studies suggest that structural cells such as epithelial cells that also express TSLP receptor could respond to TSLP. [21] We therefore examined the expression of TSLPR and IL-7Rα, which form a heterodimeric receptor for TSLP, in three types of human colon cancer cell line including SW1116, SW480 and DLD-1. As shown in Figure 2A, TSLPR mRNA was detected in all three cell lines, which had higher expression levels than PBMCs from healthy donors we used as a positive control for TSLPR expression, while IL-7Rα mRNA expression levels were comparable in the colon cancer cell lines and PBMC. We next examined TSLPR expression at protein levels in all three cell lines by immunostaining and flow cytometry. Figure 2B showed TSLPR expression on the surface and in the cytoplasm of all colon cancer cells investigated. This was further confirmed by flow cytometric analysis (Figure 2C). Moreover, we noted that SW1116 expressed TSLP at the highest level. We next examined TSLPR expression in tumor and tumor-surrounding tissues from patients with colon cancer. TSLPR mRNA expression was comparable in tumor and tumor-surrounding tissues (Figure 2D). Furthermore, TSLPR immunostaining showed abundant TSLPR-positive cells, which were primarily located in epithelium and infiltrating cells of normal mucosa in tumor-surrounding tissues. Similarly abundant TSLPR-positive staining was also detected in tumor tissues (Figure 2E). Taken together, these results demonstrate that human colon cancer cells and tumor tissues express the TSLPR and could respond to TSLP.

### Exogenous TSLP promotes the apoptosis of colon cancer cells in a TSLPR-dependent manner, but has no effect on their proliferation *in vitro*

**Figure 3:**
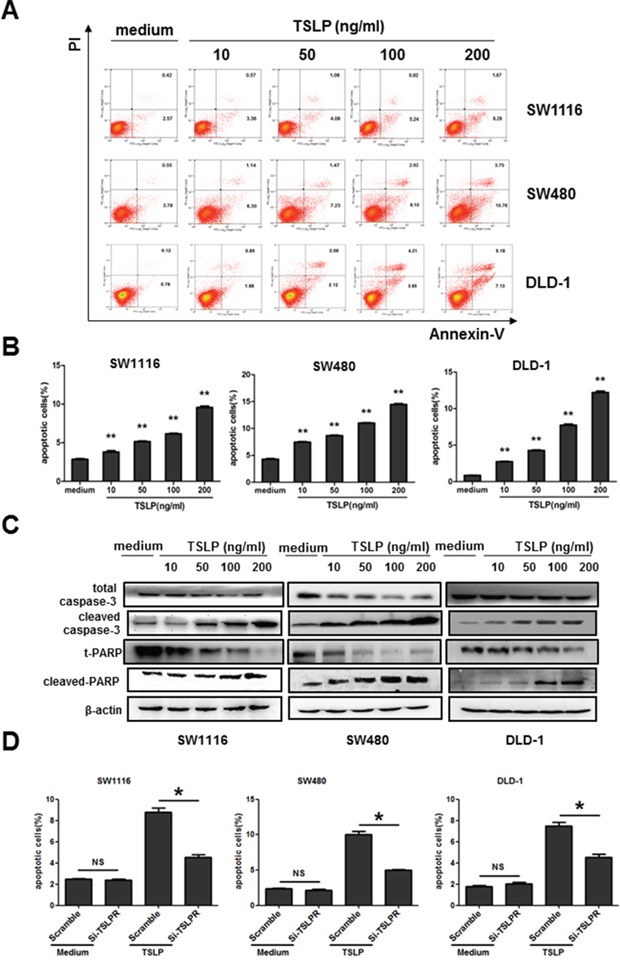
Exogenous TSLP treatment promotes apoptosis of colon cancer cells **A.** Representative data of flow cytometric analysis of Annexin V-FITC/PI double-staining apoptotic cells in three colon cancer cell lines treated with TSLP at indicated concentrations for 48 h. **B.** The percentages of apoptotic cells in TSLP-treated colon cancer cells. Columns and error bars are representatives of mean±SEM of triplicate in one experiment. Similar results were obtained in three independent experiments. **P<0.01 *versus* medium group. **C.** Western blotting analysis of protein levels of total and cleaved caspase3, t-PARP, and cleaved-PARP in TSLP-treated colon cancer cells at indicated concentrations for 48 h. β-actin was used as the control. **D.** TSLPR was specifically knocked down by siRNA in all three colon cancer cells. The average percentages of Annexin V-FITC^+^ apoptotic cells in TSLP-treated colon cancer cells were determined by flow cytometric analysis. *P<0.05 NS=no significant.

We next investigated the biological effects of TSLP on colon cancer cells by adding exogenous TSLP to the cell culture of three colon cancer cell lines at increased concentrations for 48 h. We found that the proliferation of cancer cells at 48 h was not affected by adding TSLP at all concentrations investigated as determined by BrdU Cell Proliferation Assay Kit. (Supplementary Figure S1). We next examined whether TSLP influenced the apoptosis of colon cancer cells by Annexin V-PI double staining. TSLP treatment for 48 h significantly promoted the percentages of Annexin V^+^ apoptotic cells in the culture of SW1116, SW480 and DLD-1 cells in a dose-dependent manner (Figure 3A and 3B), although higher concentrations of TSLP (100 ng/ml and 200 ng/ml) were needed to significantly promote apoptosis of colon cancer cells after 24 h incubation (Supplementary Figure S2). To further confirm the apoptosis-promoting effect of TSLP, we examined whether TSLP was able to activate caspase 3 and the downstream PARP, both of which play critical roles in cell apoptosis [22], by western blotting. We found that TSLP treatment led to markedly increased protein levels of cleaved caspase-3 and cleaved-PARP in a dose-dependent manner (Figure 3C). To assess the role of TSLPR signaling in TSLP-induced cancer cell apoptosis, we knocked down TSLPR expression in all three colon cancer cell lines by small interfering RNA (siRNA). Scramble control siRNA transfection had no effect on the apoptosis of cancer cells (Supplementary Figure S2A and Figure 3D). TSLPR knockdown significantly impaired the ability of TSLP to promote cancer cell apoptosis, as significantly lower percentages of Annexin V^+^ apoptotic cells were detected in the cultures of cancer cells transfected with TSLPR siRNA than those with scramble siRNA after 48 h following TSLP treatment (100 ng/ml) (Figure S3A and Figure 3D). Similarly, we found that TSLP had no effect on the apoptosis of a human pancreatic carcinoma cell line MIA PaCa-2 that does not express TSLPR (Supplementary Figure S3B and C). Taken together, these results demonstrate that TSLP is able to promote the apoptosis of colon cancer cells in a TSLPR-dependent manner.

**Figure 4:**
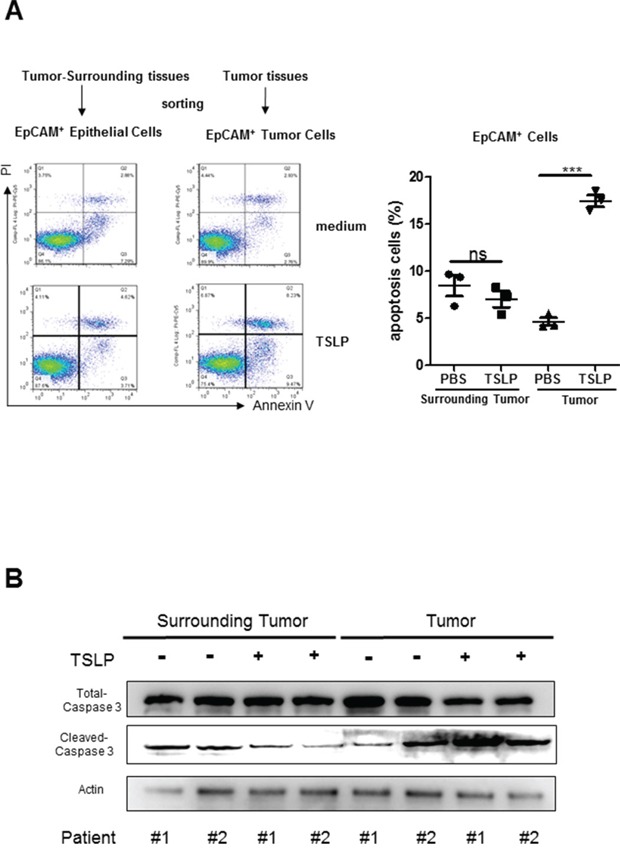
TSLP preferentially promotes the apoptosis of primary colon cancer cells EpCAM^+^ cells were from FACS sorted dissociated tumor tissues (as primary colon cancer cells) or tumor-surrounding tissues (as non-transformed colonic epithelial cells) from two patients with colon cancer. **A.** Representative data of flow cytometric analysis of Annexin V-FITC/PI double-staining apoptotic cells and the percentages of AnnexinV^+^ apoptotic cells in primary colon cancer cells and non-transformed colonic epithelial cells treated with or without TSLP. Columns and error bars are representatives of mean±SEM of triplicate in one experiment. Similar results were obtained in two independent experiments. **B.** Western blotting analysis of protein levels of total and cleaved caspase-3. β-actin was used as the control.

To confirm the apoptosis-promoting effect of TSLP on primary colon cancer cells, we FACS sorted EpCAM^+^ cells from tumor tissues from patients with colon cancer and treated them with TSLP (100 ng/ml) for 48 h. As shown in Figure 4A and 4B, TSLP treatment significantly promoted the percentages of Annexin V^+^ apoptotic cells in the culture and markedly increased protein levels of cleaved caspase-3. Since previous study showed that exogenous TSLP treatment induced anti-apoptotic BCL2 expression by murine intestinal epithelial cell line mICcl2, indicating a possible anti-apoptotic effect of TSLP [23], we next sorted EpCAM^+^ cells from tumor-surrounding tissues to examine the effect of TSLP on non-transformed human colonic epithelial cells. We found that TSLP treatment mildly decreased the percentages of Annexin V^+^ apoptotic cells accompanied by decreased protein levels of cleaved caspase-3 (Figure 4A and 4B). Taken together, these results demonstrate that TSLP is able to promote the apoptosis of primary colon cancer cells, but not non-transformed colonic human epithelial cells.

### The apoptosis-promoting effect of TSLP involves both extrinsic and intrinsic apoptosis pathways

**Figure 5:**
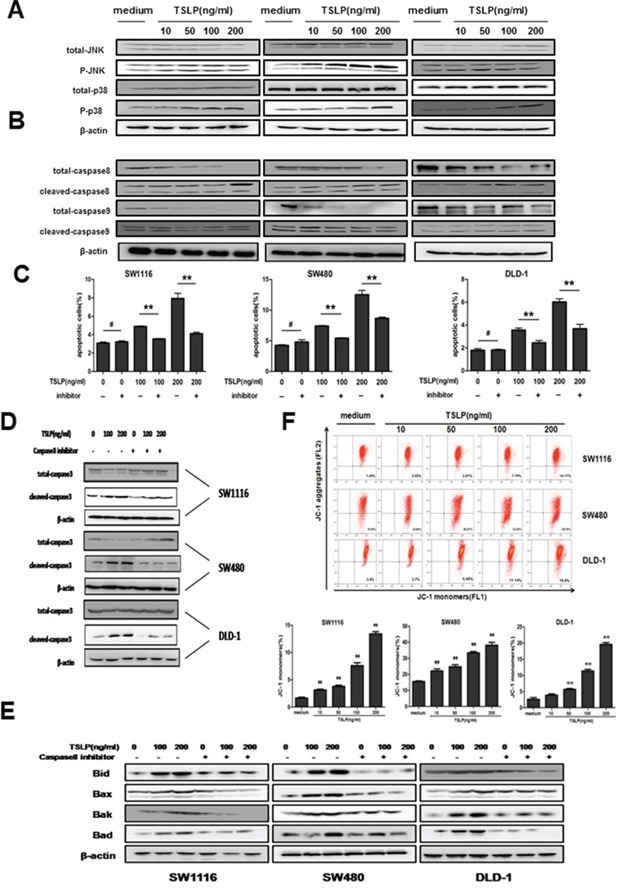
TSLP activates both extrinsic and intrinsic apoptosis pathway, and the apoptosis-promoting effect of TSLP is largely dependent on caspase-8-initiated extrinsic pathway **A** and **B.** Western blotting analysis of protein levels of total and phosphorylated MARKs (JNK and p38) **(A)** as well as total and cleaved caspase-8 and caspase-9 **(B)** in TSLP-treated colon cancer cells at indicated concentrations for 48 h. β-actin was used as the control. **C.** The percentages of Annexin V-FITC^+^ apoptotic cells in TSLP-treated colon cancer cells with or without caspase-8 inhibitor. Columns and error bars are representatives of mean±SEM of triplicate in one experiment. Similar results were obtained in three independent experiments. **D.** Western blotting analysis of protein levels of total and cleaved caspase-3 in TSLP-treated colon cancer cells with or without caspase-8 inhibitor. **E.** Western blotting analysis of protein levels of Bid, Bax, Bak, and Bad in TSLP-treated colon cancer cells at indicated concentrations for 48 h with or without caspase8 inhibitor. β-actin was used as the control. **F.** The alteration in mitochondrial membrane potential was determined by flow cytometric analysis of JC-1 staining of TSLP-treated colon cancer cells at indicated concentrations for 48 h. JC-1 is a type of fluorescence probe, which exists as JC-aggregates emitting red fluorescence detected at FL-2 channel when cells are alive and as JC-monomers emitting green fluorescence detected at FL-1 channel when early apoptosis occurs. Columns and error bars are representatives of mean±SEM of triplicate in one experiment. Similar results were obtained in three independent experiments. **P<0.01 *versus* medium group.

We next investigated the signaling pathway by which TSLP induced the apoptosis of colon cancer cell. It was reported that in airway smooth muscle cells, TSLP activates downstream MARK pathways including JNK and p38 [16, 23], which are involved in cell apoptosis as stress-inducible molecules. We performed western blotting to assess the phosphorylation levels of MARKs in TSLP-stimulated colon cancer cells. As shown in Figure 5A, TSLP induced marked phosphorylation of JNK and p38 in a dose-dependent manner. It is known that apoptosis can be initiated through mitochondrial (intrinsic) pathway or receptor (extrinsic) pathway [24]. We therefore examined whether TSLP was able to activate caspase-8 and caspase-9, which are critical effector molecules that initiate extrinsic and intrinsic apoptosis pathways, respectively [24]. Markedly increased protein levels of cleaved caspase-8 and caspase-9 were detected in TSLP-treated colon cancer cells, suggesting the involvement of both pathways (Figure 5B). To further evaluate the relative contribution of extrinsic apoptosis pathways to the apoptosis-promoting effect of TSLP on colon cancer cells, colon cancer cells were treated with specific inhibitor of caspase-8 simultaneously with TSLP stimulation. As shown in Figure 5C, inhibition of caspase-8 almost completely reversed the apoptotic levels of SW1116, SW480 and DLD-1 cells after TSLP stimulation at 100 ng/ml to the baseline levels, and significantly decreased apoptosis of colon cancer cells after TSLP stimulation at 200 ng/ml. Moreover, greatly decreased protein levels of cleaved caspase-3 were also observed in all three colon cell lines after caspase-8 inhibitor treatment (Figure 5D). These results suggest that the apoptosis-promoting effect of TSLP on colon cancer cells is largely dependent on the activation of the extrinsic pathway.

In addition to directly cleaving and activating caspase-3, activated caspase-8 can alternatively causes cleavage of Bid, a pro-apoptotic Bcl-2 family protein, which translocates to mitochondria, inducing cytochrome c release, sequentially activating caspase-9 and -3, [25] We therefore attempted to investigate whether TSLP treatment could influence the expression of proteins in mitochondria-dependent apoptotic pathway. TSLP treatment for 48h markedly increased Bid protein levels as well as other Bcl-2 family proteins that are involved in the mitochondrial apoptosis pathway including Bax, Bak and Bad in a concentration-dependent manner (Figure 5E). Moreover, inhibition of caspase-8 could reverse the up-regulation of those Bcl-2 family proteins, indicating that mitochondria-dependent apoptotic pathway was further promoted by extrinsic apoptosis pathways mediated by TSLP (Figure 5E). To further confirm that the mitochondrial apoptosis pathway was also involved in the apoptosis-promoting effect of TSLP, we used JC-1 staining to estimate transformation of mitochondrial membrane potential, which occurs in the early stage of mitochondria-dependent apoptosis [26]. TSLP treatment significantly increased the percentage of apoptotic colon cancer cells that showed green fluorescence emitted by JC-monomers in a concentration-dependent manner (Figure 5F). Taken together, these results suggest that TSLP-induced apoptosis is primarily initiated through the extrinsic pathway, which further activates the mitochondrial apoptosis pathway.

### TSLP administration inhibits colon tumor growth *in vivo*

**Figure 6:**
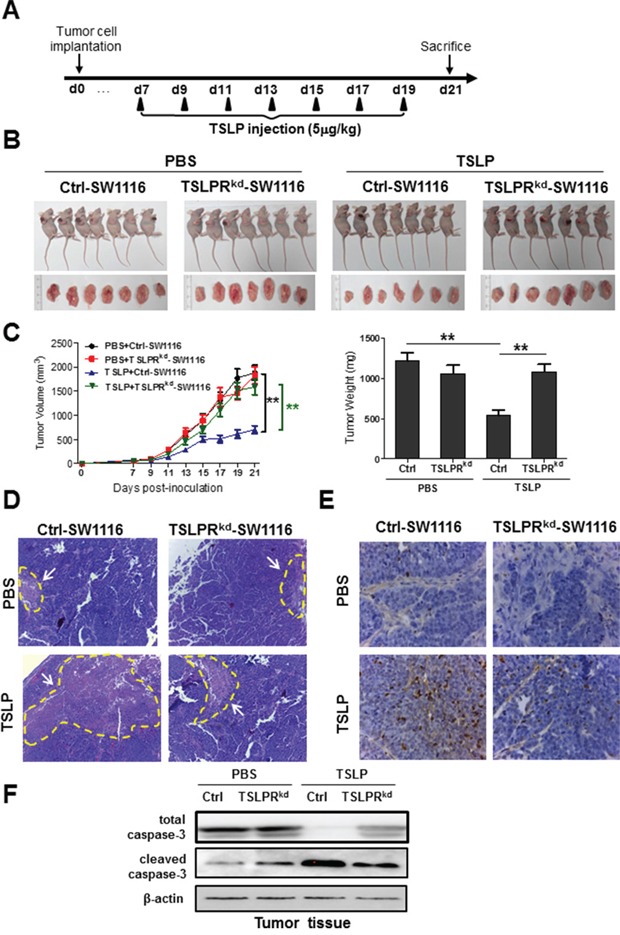
Administration of exogenous TSLP inhibits tumor growth in a xenograft mouse model of human colon cancer SW1116 and TSLPR^kd^-SW1116 cells were subcutaneously injected, respectively, into nude mice at day 0. **A.** Experimental procedure used to administer TSLP to nude mice bearing colon tumors. PBS-0.1%BSA was used as control. **B.** Tumor-bearing mice and tumors excised from each group at day 21. **C.** Tumor volume and tumor weight at day 21 were measured. **p<0.01 n=7. Data are presented as mean±SEM. **D and E.** Tumor sections from each group were stained with H&E to show necrotic area **(D)** and with TUNEL kit to show the apoptotic cells **(E)**. **F.** Western blotting analysis of protein levels of total and cleaved caspase-3 in tumor tissue lysates.

We next investigated the effect of TSLP on tumor growth in the xenograft mouse colon cancer model. SW1116 cells were used as they expressed highest levels of TSLPR and had a higher propensity for tumor formation after subcutaneous injection than other two colon cancer cell lines. TSLP or PBS as control was injected into surrounding sites of the tumor every other day starting from day 7 when tumor was palpable on mice flank until day 19 (Figure 6A). There were significant decreases in the volume and the weight of tumors from TSLP-treated group compared with those from control mice, indicating the inhibitory effect of TSLP on tumor growth (Figure 6B-6C). Histological examination revealed greater necrotic areas in tumors from TSLP-treated group than those from control mice (Figure 6D). To further determine whether TSLP treatment induced apoptotic response *in vivo*, TUNEL staining of tumor sections was performed. There were much more TUNEL-staining apoptotic cells in the tumor sections from TSLP-treated mice than those from control mice (Figure 6E). Consistently, western blotting analysis showed increased protein levels of cleaved caspase-3 in tumors from TSLP-treated mice compared with those from control mice (Figure 6F). To examine whether TSLPR signaling is required for the inhibitory effect of TSLP on tumor growth, TSLPR was knocked down in SW1116 cells (denoted as TSLPR^kd^-SW1116) by short-hairpin RNA (shRNA). TSLPR knockdown had no effect on SW1116 tumor growth *in vivo* (Figure 6B-6C). Importantly, TSLP treatment failed to inhibit TSLPR^kd^-SW1116-derived tumor growth (Figure 6B-6C), which was accompanied by similar tumor necrotic areas and apoptotic responses to those in control group without TSLP treatment (Figure 6D-6F). Taken together, these results demonstrate that TSLP is able to inhibit tumor growth in a xenograft mouse model of colon cancer, which is dependent on TSLPR signaling in cancer cells.

## DISCUSSION

We here demonstrate that TSLP was down-regulated in human colon tumors, which negatively correlates with the advanced stage of this disease. Moreover, administration of exogenous TSLP is able to promote the apoptosis of human colon cancer cells, and inhibit colon tumor growth in a xenograft mouse model of colon cancer in a TSLPR-dependent manner.

In contrast to increased TSLP expression reported in breast cancer and pancreatic cancer, we found that TSLP expression levels was significantly down-regulated in colon tumors by using two sets of public dataset and the surgical specimens we collected from patients of colon cancer. More importantly, we found that the patients with tumoral TSLP expression at the lowest levels had the most advanced diseases, indicating a tumor-suppressing role of TSLP in colon cancer. Various factors have been reported to regulate TSLP expression under different pathological conditions, among which miR-375 was shown to up-regulate TSLP in intestinal epithelial cells following helminth infection [27]. Recent study also showed that miR-375 was the single most down-regulated miRNA in rectal cancer [28]. Interestingly, we found that TSLP positively correlated with miR-375 expression in colon tumors tissues (Supplementary Figure S4), implying a possible involvement of miR-375 in down-regulated TSLP expression in colon cancer cells.

Extensive attention has been focused on the regulatory role of TSLP in immunity. TSLPR was recently demonstrated to be expressed in some non-hematopoietic cells such as human airway smooth muscle cells and IECs [12, 16]. Here we for the first time showed that colon cancer cells expressed TSLPR, suggesting that TSLP might directly act on colon cancer cells. This was further supported by our findings that TSLP significantly promoted the apoptosis of all three colon cancer cell lines. Moreover, we also confirmed the apoptosis-promoting effect of TSLP on primary colon cancer cells isolated from human colon tumors. In contrast, we found a mild anti-apoptotic effect of TSLP on non-transformed human colonic epithelial cells, which is consistent with a previous study [23]. Actually, similar to TSLP, another important protein TRAIL also preferentially induced apoptosis in cancer cells [29]. The preference of TSLP to promote the apoptosis of colon cancer cells could be due to aberrant signaling networks in cancer cells which may cause different signaling pathway mediated by TSLP. Indeed, increasing evidence has demonstrated a complex regulatory frame work for TSLP signaling pathway depending on different cell types. For example, activation of STAT5 and STAT3 has been extensively reported in TSLP-stimulated lymphocytes. In contrast, TSLP could activate MAPK (ERK and p38) but not STAT3 and STAT5 in human eosinophils [30], and MAPKs (ERK, p38 and JNK) and STAT3 but not STAT5 in human airway smooth muscle cells [16]. We found that TSLP activated MAPKs (JNK and p38) and STAT5, but, interestingly, down-regulated phosphorylation of STAT3 in colon cancer cells (Supplementary Figure S5). Accumulating data showed that STAT3 inhibition could promote the apoptosis of tumor cells [31–33]. Thus, down-regulation of STAT3 phosphorylation strongly supported our findings that TSLP promoted the apoptosis of cancer cells. It would be interesting to study specific mechanisms underlying TSLP-TSLPR signaling that influence tumor development in different cell types in the future.

TSLP was previously reported to promote lymphocyte survival accompanied by increased Bcl-2 expression [34, 35], suggesting that TSLP could affect mitochondria-dependent intrinsic apoptosis pathway. In accordance with it, our data demonstrated that TSLP up-regulated the expression of proteins that mediates mitochondria-dependent apoptosis [36] and altered mitochondrial membrane potential which occurs in mitochondria-dependent early cell apoptosis [37]. Furthermore, we demonstrated that extrinsic apoptosis pathway was involved in apoptosis-promoting effect of TSLP, as cleaved caspase-8 was greatly increased in TSLP-treated colon cancer cells and caspase-8 inhibitor markedly decreased cancer cell apoptosis. Considering that the biological function of TSLP is mediated by TSLPR and caspase-8 activation could result in alternatively activated intrinsic apoptosis pathway [25], it is very likely that TSLP-induced apoptosis of colon cancer cells is largely dependent on the extrinsic apoptosis pathway, albeit involves both extrinsic and intrinsic pathway.

The ability of TSLP to promote the apoptosis of colon cancer cells was further confirmed by using xenograft mouse model of colon cancer, underscoring the anti-tumor effect of TSLP. This seemed in contrast to the tumor-promoting effect of TSLP reported in pancreatic cancer and breast cancer. The pro-tumor role of TSLP in those studies has been attributed to its ability to promote the inflammatory Th2 microenvironment [13, 15]. However, Th2 cells are not a prognostic factor for colon cancer [18], suggesting that the role of TSLP in colon cancer could be independent of its effect on Th2 immunity. Moreover, our *in vivo* results demonstrated that tumor-inhibitory effect of TSLP was, at least partly, mediated by TSLPR expressed in colon tumor cells. Thus, the discrepancy could be explained by the different type of target cell of TSLP and the relative contribution of TSLP-regulated immune response to the growth of different tumor types. However, we still cannot exclude the possibility of TSLP directly acting on immune cells to regulate the growth of colon cancer. Further investigation needs to explore the immunoregulatory effect of TSLP on colon cancer by using cancer model of immunocompetent mice and TSLP or TSLPR-deficient mice. Notably, a tumor-suppressive effect of TSLP-TSLPR signaling recently has been demonstrated in mouse models of skin cancer by promoting a T cell-mediated antitumor response and inhibiting skin accumulation of immunosuppressive Gr-1^+^ myeloid cells [38, 39]. Together with our data, these results highlighted the context-dependence of TSLP signaling in cancer.

In summary, our study reveals a novel anti-tumor function of TSLP by inducing the apoptosis of colon cancer cells in a TSLP-dependent way, and decreased TLSP levels could contribute to the growth of colon tumor. Thus, our study suggests TSLP could be a potential therapeutic target for colon cancer, particularly those with compromised immune system after long-term chemotherapy or radiotherapy.

## MATERIALS AND METHODS

### Mice and reagents

Four-week-old female nude mice were purchased from Chinese Academy of Science (Shanghai) and housed in a pathogen-free facility. Experimental procedures were in accordance with the Animal Care and Use Committee at Fudan University. Recombinant human TSLP, rabbit anti-human TSLP antibody and goat anti-human TSLPR antibody applied for immunocytochemistry were purchased from R&D. FITC-conjugated anti-human TSLPR antibody were purchased from eBioscience.

### Patients and specimens

Totally forty colon cancer patients, who were diagnosed by colonoscopy and biopsy pathology, were recruited in this study in Huashan Hospital, Fudan University, Shanghai, China. Patients that suffered from other cancers, severe dysfunction of organs as heart, liver and kidney, autoimmune disease were excluded. These patients averagely aged 63 including 27 males and 13 females, and were divided into four groups according to criteria of Duke's classification: six cases of stage A, sixteen of stage B, twelve of stage C and six of stage D. The details of tumor characteristics are shown in Supplementary Table S1. All patients received tumor-excision operation. The intestinal specimens were separated into tumor and tumor-surrounding tissues for further examination. The study was approved by the ethics committee of Huashan Hospital, Fudan University. All patients signed an informed-consent agreement. Freshly resected tumor tissues and colon tissues were obtained from patients between May and July 2015 at Huashan Hospital, Fudan University, Shanghai, China.

### Measurement of TSLP expression in human colon cancer tissue by ELISA

Tumor or tumor-surrounding tissues were homogenized in 500 ml ice-cold PBS containing a protease inhibitor cocktail (Sigma-Aldrich), and total protein contents were measured by Bicinchoninic acid (BCA) kit (Sangon Biotech, China). The concentrations of TSLP were measured using an ELISA Kit for human TSLP (R&D Systems). The TSLP concentration was normalized to total protein content.

### Cell culture

The colon cancer cell lines SW1116 (catalogue number: CCL-233), SW480 (catalogue number: CCL-228) and DLD-1 (catalogue number: CCL-221) were obtained from American Type Culture Collection (ATCC). Cells (1×10^5^ cells/ml) were treated with serial concentrations of exogenous TSLP for 48h. In some experiments, caspase8 inhibitor Z-IETD-FMK (Calbiochem) at a dose of 10 nM was added simultaneously with TSLP, then cells were harvested for flow cytometry and western blotting.

### Flow cytometry

Colon cancer cells were stained with FITC-conjugated human anti-TSLPR antibody and with annexin-V and PI kit (BD Pharmingen). For the analysis of mitochondrial membrane potential, cancer cells were incubated with JC-1 reagent (Beyotime, China) according to the manufacturer's instruction. Cells were analyzed by CyAn™ ADP Analyzer (Beckman Coulter). All data was processed using Summit V5.2.0 program.

### FACS sorting

Normal colon tissues and tumor tissues were cut into small pieces (∼1 mm3), then digested by collagenase IV and Dispase II (both in 1 mg/ml, Sigma) at 37°C for 1 hr. Single cell suspension was obtained by filtering through 40 mm meshes and washed by 1x HBSS (Hank's Balanced Salt Solution). Cells were cultured by Epithelial culture medium (Catalogue #2951, Sciencell). Primary colon cancer cells and non-transformed colonic epithelial cells were sorted based on EpCAM expression by Moflo (Beckman Coulter).

### RT-PCR

Total RNA was extracted from tumor or tumor-surrounding-tissues of patients with colon cancer or colon cancer cell lines using TRIzol (Invitrogen), and cDNA was generated using PrimeScriptRT Master Mix (TaKaRa Biotechnology). For qRT-PCR, Power SYBR Green Master kit (ABI) and Applied Biosystems 7500 were used. The relative expression of target genes was calculated using the 2ΔC(t) method. The primer sequences of genes were shown in Supplementary Table S2.

### RNA interference

The siRNA sequence targeted TSLPR (crlf2, GenBank ID: NM_022148) and the scramble siRNA sequence were designed and synthesized by GenePharma (Shanghai, CHINA). The sequence for si-TSLPR was 5′-GCGGTGATGTGGTCACAAT-3′. For *in vitro* experiments, siRNAs were transfected into three colon cancer cell lines by lipofectamineiMAX (Invitrogen, USA) according to the manufacturer's instructions. For *in vivo* experiment, the Sh-RNAs vector targeted TSLPR or scrambled control vector (GenePharma, Shanghai) were transfected into SW1116 by lipofectamine 3000 (Invitrogen, USA). The TSLPR targeted sequence was: 5′-CAAACCAAAGCTGTCCAAA-3′). The efficiency of RNA interference was determined by qRT-PCR.

### Xenograft model of human colon cancer

SW1116 or TSLPR^kd^-SW1116 cells (2×10^6^ cells in 100 ml DMEM solution per mouse) were subcutaneously injected into the flanks of mice. When the tumors were palpable on day 7, TSLP (5 μg/kg in 10ml PBS-0.1%BSA) or PBS-0.1%BSA as control was administered around the tumor site every other day for two weeks followed by measuring tumor volume with calipers every 2 days. The tumor volume was calculated by (length X width^2^) X 0.5. At day 21, all mice were sacrificed and tumors were excised for further examination.

### Immunohistochemistry and histology analysis

For the detection of TSLPR expression by colon cancer cells, the coverslips were put into 6-well plate before the seeding of colon cancer cells, the cell climbing slices were taken out and fixed in 4% paraformaldehyde. Paraffin-embedded human colon sections and climbing slices were incubated with rabbit anti-human TSLP antibody and goat anti-human TSLPR antibody (1:100 dilution, R&D), respectively. The secondary antibody used was HRP-conjugated goat anti-rabbit IgG and mouse anti-goat IgG (1:200 dilution, ebioscience), respectively. Slices added with PBS instead of the primary antibodies were used as negative controls. The intensity of TSLP staining was evaluated in a blind manner by two independent investigators and graded by a 5-scale system (0, no signal; 1, weak; 2, moderate; 3, strong; 4, very strong; and 5, extremely strong). Paraffin-embedded sections of tumor excised from the mice were stained with Hematoxylin & Eosin. The necrotic areas were examined under microscope. Detection of fluorescence binding to 3′-OH of DNA fragments was performed with the TUNEL *in situ* cell death detection kit-POD (Roche), according to the manufacturer's instruction.

### Western-blotting

Protein extracts were prepared by lysing colon cancer cells or tumor tissues. Total protein concentrations were determined by BCA assay (Sangon Biotech, China). Aliquots of cell extractswere separated by SDS-PAGE and transferred to a PVDF membrane (Amersham Pharmacia Biotech) by electroblotter (Bio-Rad). Membranes were blocked and incubated overnight with primary antibodies against PARP, cleaved-PARP, total and cleaved-caspase3, total and cleaved-caspase8, total and cleaved-caspase9, total and p-JNK, total and p-p38MAPK (these antibodies used at 1:2000dilution), Bid, Bad, Bax, Bak, t-STAT3 (1:1000dilution), p-STAT3, t-STAT5, p-STAT5 (these antibodies used at 1:1000 dilution), and β-actin(1:2000dilution) at 4°C overnight. Then the membranes were incubated with HRP-conjugated anti-rabbit or anti-mouse IgG (1:3000 dilution) for one hour at RT. All these antibodies were purchased from Cell Signaling Technology Inc. Membranes were subsequently incubated with ECL kits (Pierce/Thermo Fisher Scientific) and developed on chemiluminescence imaging system (ImageQuant™ LAS 4000).

### Statistical analysis

For analyzing TSLP expression, differences between two groups and among three or more groups were assessed by paired student t-test and one-way ANOVA analysis, respectively. For analyzing apoptosis rate, differences between two groups and among three or more groups were assessed by Mann-Whitney U and Kruskal-Wallis H analysis, respectively. For analyzing tumor weight and volume of nude mice, differences betweentwo groups were assessed by unpaired student t-test. All procedures were operated using spss17.0 software and values of p<0.05 were considered statistically significant.

## SUPPLEMENTARY FIGURES AND TABLES


