# Supplementary material for: Thymic stromal lymphopoietin (TSLP) inhibits human colon tumor growth by promoting apoptosis of tumor cells
Source: Oncotarget. 2016 Feb 23;7(13):16840–54. doi: 10.18632/oncotarget.7614 (PMC4941354; doi:10.18632/oncotarget.7614)
Supplement: Supplementary file 1 [file oncotarget-07-16840-s001.pdf]

SUPPLEMENTARY FIGURES AND TABLES

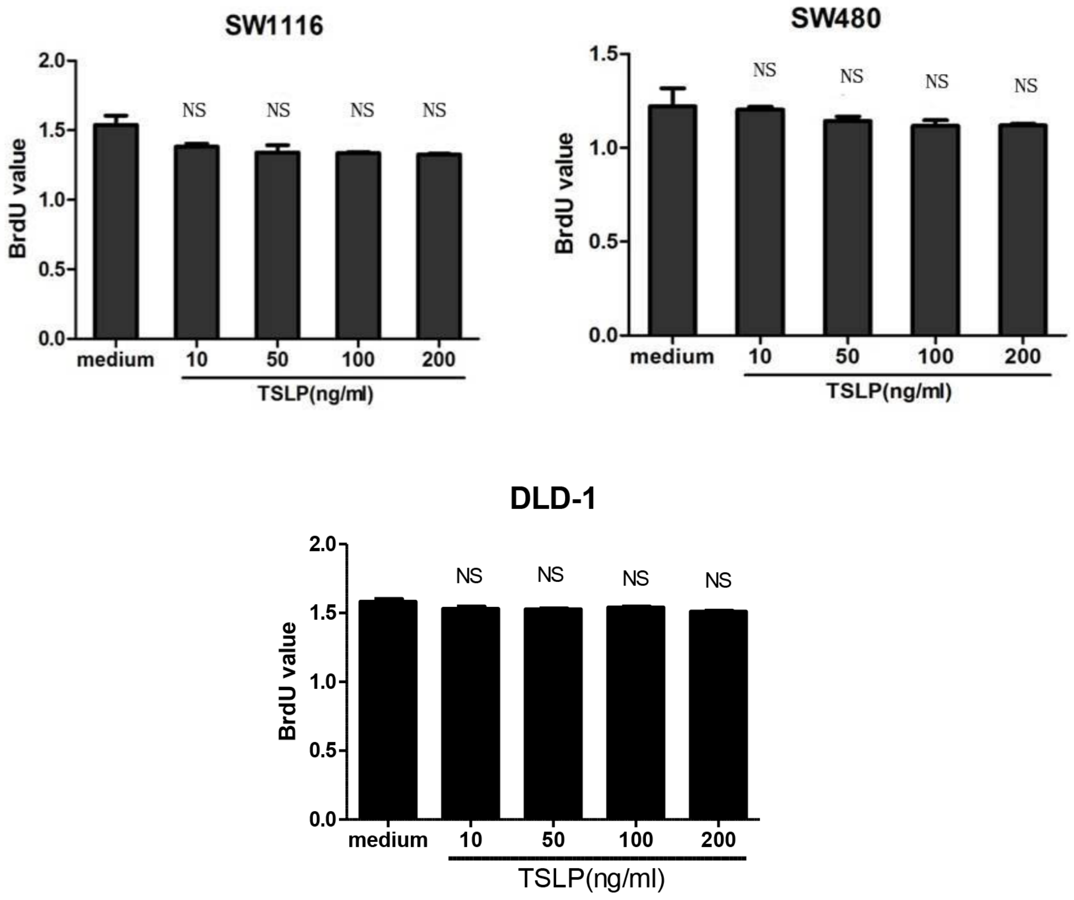

Supplementary Figure S1: Exogenous TSLP treatment does not influence the proliferation of colon cancer cells. Cells were stimulated with TSLP at indicated concentrations for 48 h followed by incubating with BrdU for 30 min, and the BrdU values were measured. Columns and error bars are representatives of mean±SEM of triplicate in one experiment. Similar results were obtained in three independent experiments. NS=no significant.

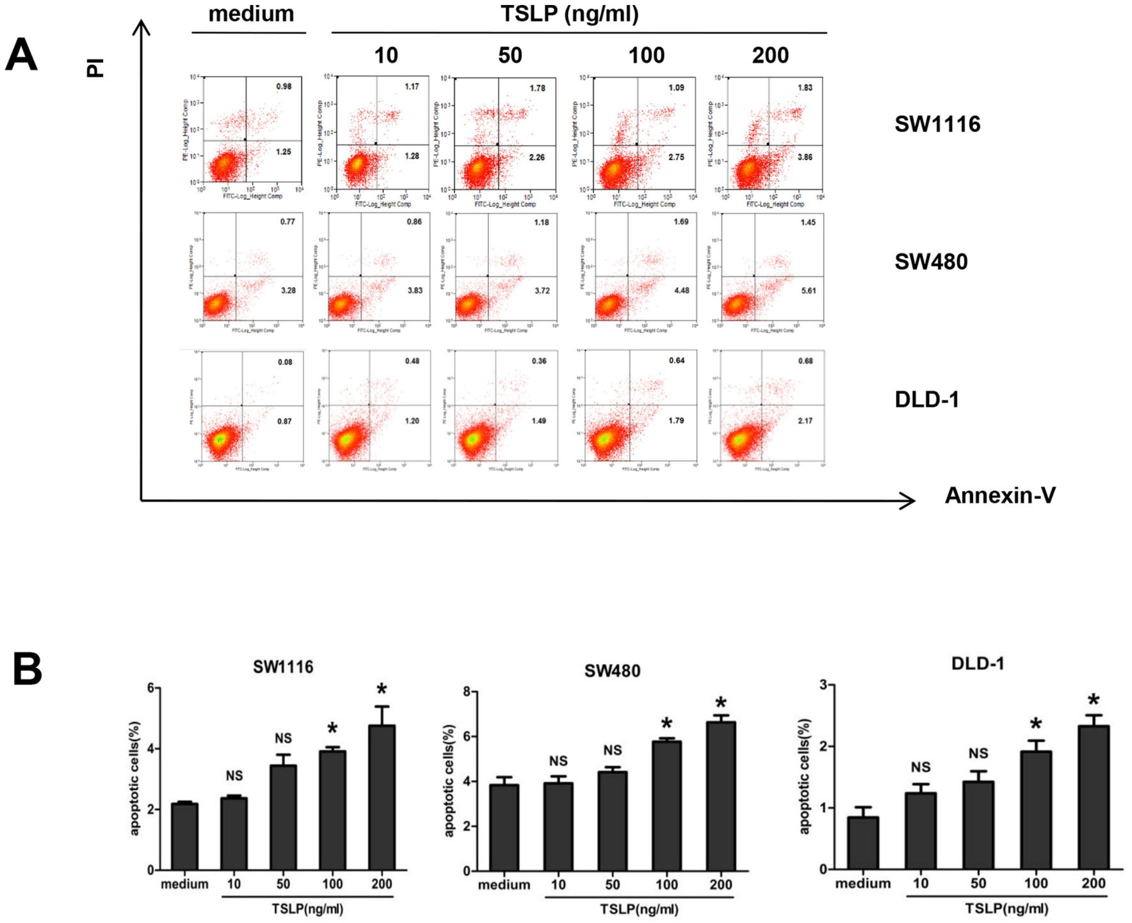

Supplementary Figure S2: TSLP treatment for 24 h promotes apoptosis of colon cancer cells at higher concentrations. **A.** Representative data of flow cytometric analysis of Annexin V-FITC/PI double-staining apoptotic cells in three colon cancer cell lines treated with TSLP at indicated concentrations for 24 h. **B.** The percentages of apoptotic cells in TSLP-treated colon cancer cells. Columns and error bars are representatives of mean±SEM of triplicate in one experiment. Similar results were obtained in three independent experiments. NS=no significant ; *P<0.05 *versus* medium group.

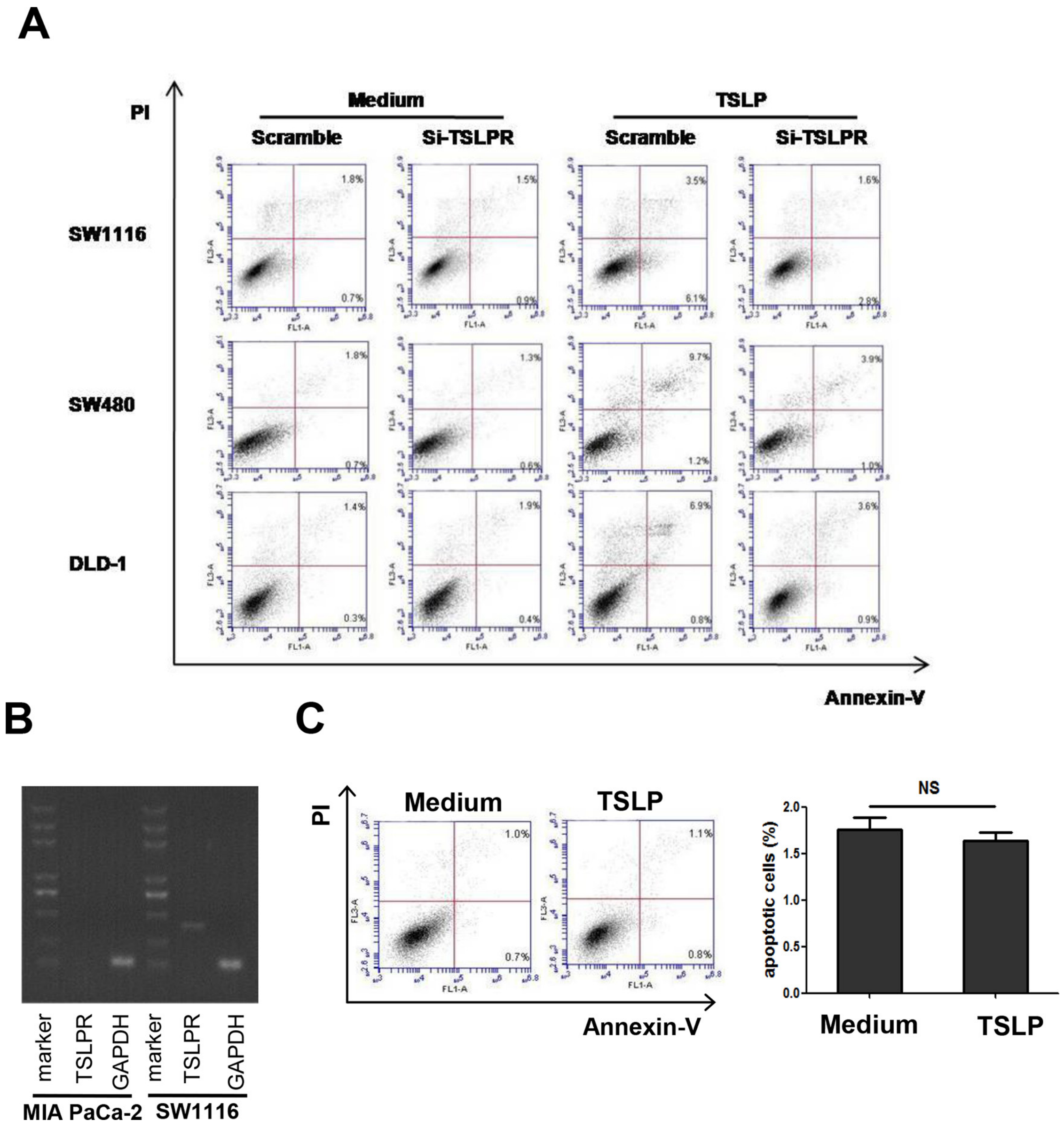

Supplementary Figure S3: The apoptosis-promoting activity of exogenous TSLP is mediated by TSLPR signaling. **A.** Representative data of flow cytometric analysis of Annexin V-FITC/PI double-staining apoptotic cells in three colon cancer cell lines in which TSLPR was down-regulated by siRNA at 48 h after TSLP treatment (100 ng/ml). **B.** TSLPR mRNA expression was undetectable in MIA-Paca2. **C.** Representative data of flow cytometric analysis of Annexin V-FITC/PI double-staining apoptotic cells and the average percentages of apoptotic cells in TSLP-treated MIA-Paca2. NS=no significant.

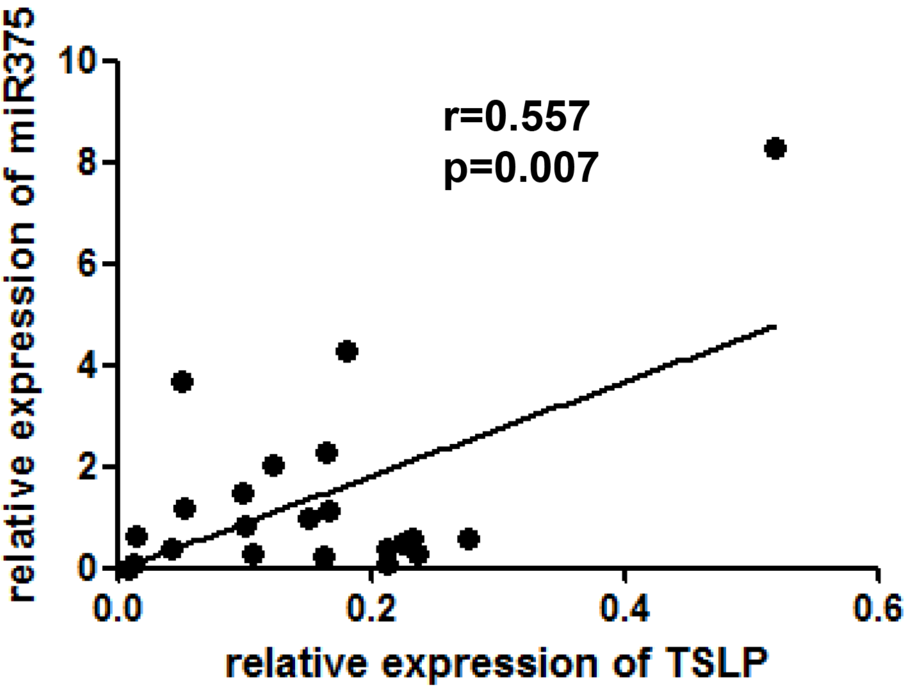

Supplementary Figure S4: TSLP positively correlates with miR-375 expression in human colon tumor tissues. Gene expression of TSLP and miR-375 was examined by qRT-PCR analysis. The correlation between TSLP and miR-375 gene expression was analyzed by Pearson correlation coefficient.

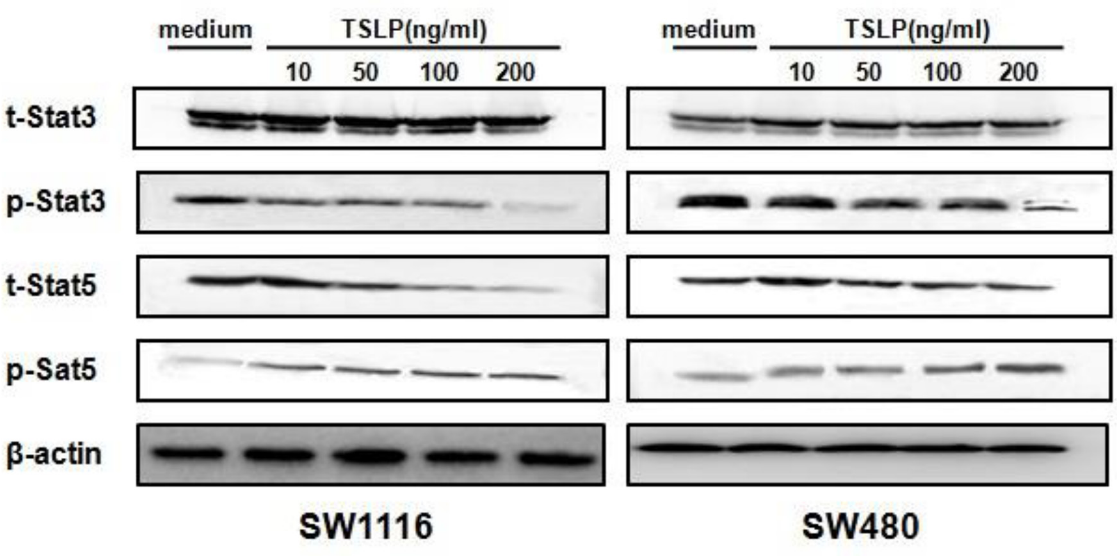

Supplementary Figure S5: TSLP treatment causes up-regulation of phosphorylated STAT5 but down-regulation of phosphorylated STAT3 levels in colon cancer cells. Western blotting analysis of protein levels of total and phosphorylated STATs (STAT3 and STAT5) in TSLP-treated colon cancer cells (SW1116 and SW480) at indicated concentrations for 48 h. β-actin was used as the control.

Supplementary Table S1: clinic characteristics of colon cancer patients

	Duke's A (n=6)	Duke's B (n=16)	Duke's C (n=12)	Duke's D (n=6)
male	4	10	8	5
age	63.5	63.3	62.4	62.1
tumor diameter(>5cm)	1	9	6	1
tumor site				
ileocecus	1	3	1	1
ascending colon	1	2	1	2
transverse colon	0	1	0	0
sigmoid colon	0	6	1	3
rectum	4	4	9	0
operation				
partial resection	2	12	6	6
mile's	2	1	4	0
Dixon	2	2	1	0
Hartmann	0	1	1	0

Supplementary Table S2: Primer sequences

TSLP	forward	5'-TATGAGTGGGACCAAAAGTACCG-3'
	reverse	5'-GGGATTGAAGGTTAGGCTCTGG-3'
TSLPR	forward	5'-GAGTGGCAGTCCAAACAGGAA-3'
	reverse	5'-ACATCCTCCATAGCCTTCACC-3'
IL-7R	forward	5'-TGGACGCATGTGAATTTATC-3'
	reverse	5'-CATTCACTCCAGAAGCCTTT-3'
GAPDH	forward	5'-CAAAAGGGTCATCATCTCTG-3'
	reverse	5'-CCTGCTTCACCACCTTCTTG-3'
